# Complete Atrioventricular Septal Defects after the Age of 40 Years

**DOI:** 10.3390/jcm10163665

**Published:** 2021-08-19

**Authors:** Susanne J. Maurer, Lorena Moosholzer, Claudia Pujol, Nicole Nagdyman, Peter Ewert, Oktay Tutarel

**Affiliations:** 1 Department of Electrophysiology, German Heart Centre Munich, TUM School of Medicine, Technical University of Munich, 80636 Munich, Germany; susanne.maurer@tum.de; 2 Department of Congenital Heart Disease and Paediatric Cardiology, German Heart Centre Munich, TUM School of Medicine, Technical University of Munich, 80636 Munich, Germany; lorenamoosholzer@hotmail.com (L.M.); claupujol@gmail.com (C.P.); nagdyman@dhm.mhn.de (N.N.); ewert@dhm.mhn.de (P.E.); 3 DZHK (German Centre for Cardiovascular Research), Partner Site Munich Heart Alliance, 80992 Munich, Germany

**Keywords:** adult congenital heart disease, atrioventricular septal defects, mortality

## Abstract

Background: There is an increasing number of adults with complete atrioventricular septal defects (cAVSD). However, data regarding older adults are lacking. The aim of this study is to analyze the outcome of adults with cAVSD over the age of 40 years. Methods: Patients with cAVSD who were ≥40 years of age at any point between 2005 until 2018 were included retrospectively. Data were retrieved from hospital records. The primary endpoint was a combination of death from any cause and unplanned hospitalizations due to cardiac reasons. Results: 43 patients (60.5% female, mean age 43.7 ± 6.0 years, genetic syndrome 58.1%) were included. At begin of follow-up, the majority of patients (*n* = 41, 95.3%) was in New York Heart Association (NYHA) class I or II. Out of the whole cohort 26 (60.5%) had undergone cardiac surgery. At baseline, at least one extracardiac comorbidity was present in 40 patients (93.0%). Median follow-up was 1.7 years (IQR 0.3–4.6). On univariate Cox analysis, NYHA class at begin of follow-up (hazard ratio: 1.96, CI 95%: 1.04–3.72, *p* < 0.05) was the only predictor for the primary endpoint. Conclusions: Significant morbidity and mortality is present in cAVSD patients over the age of 40 years. NYHA class is predictive for a worse outcome.

## 1. Introduction

Improvements in the diagnostic, as well as medical, interventional, and surgical treatment have led to an improved survival even of patients with complex forms of congenital heart defects (CHD) [1]. Complete atrioventricular septal defect (cAVSD) is one CHD for which a surgical correction was already reported in the 1950s [2]. Since then, significant advances were made in the treatment of children born with a cAVSD [3] and the number of adults with a cAVSD is, therefore, increasing [1]. An estimated survival rate of 71% at 30 years after initial cAVSD repair has been reported in one large series [4]. The estimated overall survival for the hospital survivors (excluding those with early postoperative deaths) was 80% at 30 years in the same series [4]. However, limited data are available about the clinical course of cAVSD patients over the age of 40 years, while we know that the number of adults with CHD (ACHD) over the age of 40 years are increasing in general, [5] and also in subgroups of specific CHD [6,7]. The aim of this study is to assess the clinical course of cAVSD patients over the age of 40 years and to identify risk factors for a worse outcome.

## 2. Materials and Methods

This retrospective, single-center study included all patients with a cAVSD diagnosis under follow-up at the German Heart Centre Munich who were ≥40 years of age at any time point between January 2005 and December 2018. Date of inclusion and begin of follow-up was either the 40th birthday or, if the patient was already 40 years old in the year 2005, the first visit after the 1st of January 2005, as previously described [6,7].

Only patients with a cAVSD were included. Patients with an atrioventricular septal defect (AVSD) with an isolated atrial component (also known as atrial septal defect primum, ostium primum defect, or partial AVSD) [8] were excluded. Corrected patients encompassed all with a surgical correction, while the uncorrected group included patients without surgery and those with a palliation. 

Demographic data and information on medical and surgical history were retrieved from hospital records. Symptomatic status was assessed according to the New York Heart Association classification (NYHA). Based on the results of routine transthoracic echocardiograms, left and right ventricular systolic function was graded semi-quantitatively as normal, or mildly, moderately, or severely impaired, as described previously [9]. Pulmonary hypertension was diagnosed according to current guidelines [10]. Arrhythmias encompassed any type of atrial or ventricular arrhythmia requiring treatment. Renal disease included any form of it (i.e., renal failure, glomerulonephritis, etc.). Gastrointestinal (GI) diseases included hepatobiliary disorders, for example cholecystolithiasis. Lung disease included any form of it (i.e., asthma, chronic obstructive lung disease, emphysema, etc.). Central nervous system disorders encompassed any form of it, including cerebrovascular accidents. Musculoskeletal disease included all forms of it, e.g., arthrosis. Genitourinary disorders included all forms, for example stenosis of the ureter. Eye or ear, nose and throat diseases incorporated any disease affecting these organ systems, e.g., cataract. Cyanotic patients were defined by an oxygen saturation less than 90% at rest. 

The primary endpoint was a combination of death from any cause and unplanned hospitalizations due to cardiac reasons.

Statistical analyses were performed using SPSS version 25 (IBM Corp., Armonk, NY, USA) and MedCalc version 20 (MedCalc Software, Ostend, Belgium). Continuous variables are presented as mean ± standard deviation or median (interquartile range), whereas categorical variables are presented as number (percentage). Comparison between groups was performed using the Mann–Whitney U test or Student’s *t*-test for continuous and Chi-square test for categorical variables. Univariate Cox proportional hazards analysis was used to assess the association between variables and the primary endpoint. Kaplan-Meier curves and log-rank test were used to compare event-free survival from the primary endpoint between patients in NYHA class I and patients in NYHA class II–IV, as well as patients with corrected vs. uncorrected cAVSD. All tests were performed two-sided. For all analyses, a *p*-value < 0.05 was considered statistically significant. 

## 3. Results

Altogether, 43 patients (mean age 43.7 ± 6.0 years, female 60.5%) were included. Down syndrome was present in 23 patients (53.5%), Holt Oram syndrome in 1 (2.3%), and VACTERL association in 1 (2.3%). Out of the whole cohort, 26 (60.5%) had undergone cardiac surgery at baseline, corrective in 24. Therefore, 19 patients were in the uncorrected group (17 unoperated, 2 with palliative surgery). A pacemaker was present in 6 patients (14.0%). The New York Heart Association (NYHA) class at baseline was I in 28 patients (65.1%), II in 13 (30.2%), III in 1 (2.3%), and IV in 1 (2.3%). Pulmonary arterial hypertension (PAH) was present in 13 patients (30.2%), out of these 8 were on advanced PAH therapies at baseline. 

At least one extracardiac comorbidity was present in 40 patients (93.0%) without a significant difference between patients with and without a syndrome (*p* = 0.07). Most common were gastrointestinal disorders (*n* = 12 (27.9%)). More detailed information regarding baseline characteristics including a comparison between patients with and without a syndrome is presented in Table 1.

Detailed information regarding baseline characteristics in patients with corrected vs. uncorrected cAVSD is presented in Table 2.

Data from a baseline echocardiography were available in 31 patients. Left ventricular systolic function was normal in all patients. Right ventricular systolic function was normal in 93.1% of patients, mildly reduced in 3.4%, and moderately reduced in 3.4%. The right atrioventricular (AV) valve was non-regurgitant in 6.5%, mildly regurgitant in 35.5%, moderately in 41.9%, and severely in 16.1%. The left AV valve regurgitation was mild in 46.9%, moderate in 40.6%, and severe in 6.3%. Stenosis of the left AV valve was moderate in 2.9% and severe in 2.9%.

A follow-up was not available in seven patients. During a median follow-up of 1.7 years (IQR 0.3–4.6), atrial arrhythmias occurred in 9 out of 36 patients (mainly atrial fibrillation and atrial flutter), while ventricular arrhythmias were not reported. Four patients died during follow-up. Cause of death was sepsis in two patients, while in the other two the cause of death was not available. Ten unplanned hospitalizations due to cardiac reasons occurred (heart failure *n* = 5, endocarditis *n* = 1, chest pain *n* = 2, pericardial effusion *n* = 1, arrhythmia = 1). On univariate Cox analysis, NYHA class at begin of follow-up (hazard ratio (HR): 1.96, CI 95%: 1.04–3.72, *p* < 0.05) was the only predictor for the primary endpoint (Table 3).

**Figure 1:**
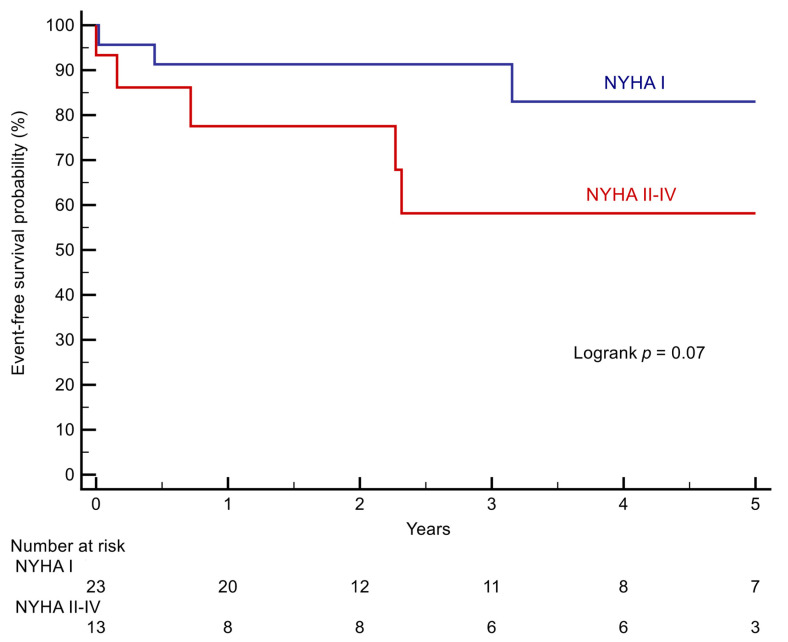
Kaplan–Meier curves stratifying patients according to NYHA class.

The difference between patients with NYHA class I and patients with NYHA II–IV at baseline approached but did not reach statistically significance (*p* = 0.07, Figure 1).

**Figure 2:**
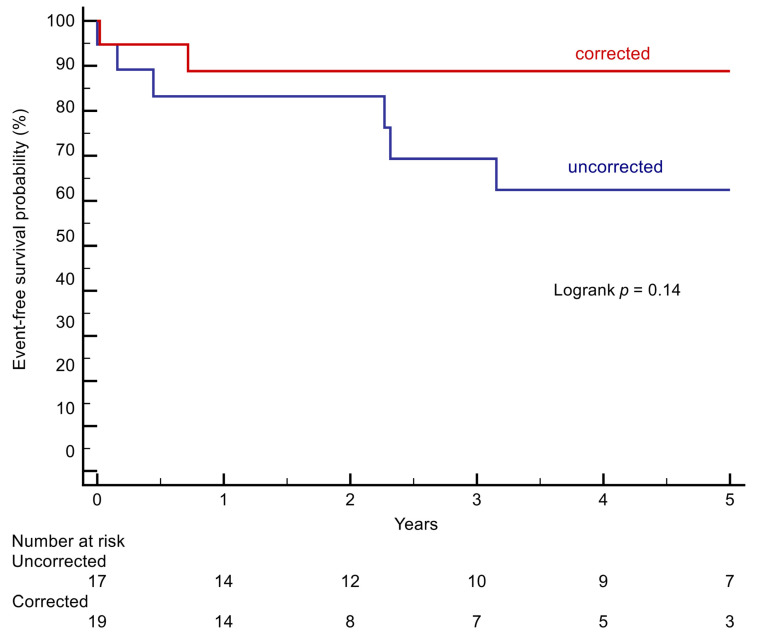
Kaplan–Meier curves comparing patients with a corrected cAVSD with uncorrected patients.

The difference between patients with corrected vs. uncorrected cAVSD was not statistically significant (*p* = 0.14, Figure 2).

## 4. Discussion

In this study, significant morbidity and mortality was present in patients with a cAVSD over the age of 40 years. Symptomatic status was predictive for the combination of death from any cause and unplanned hospitalizations due to cardiac reasons.

NYHA class emerged as a predictor for the primary endpoint. This is in difference to recent studies in patients with PAH associated with ACHD or ACHD patients with a single-ventricle physiology over the age of 40 years [6,7]. In these latter studies, comorbidities like renal disease were significant predictors. The smaller number of patients in the current study could have contributed to these differences. Bredy and colleagues recently confirmed NYHA class as an important prognostic tool in ACHD patients [11]. They included 2781 ACHD patients in their study and reported a strong relation between NYHA class and objective measures of exercise capacity, as well as the Bethesda classification [11]. Additionally, NYHA class was a strong predictor of mortality, with an 8.7-fold increased mortality risk in class III compared with class I [11]. Furthermore, functional class was a predictor of a worse clinical outcome in a large cohort of ACHD patients over the age of 60 years with a variety of underlying CHD [5]. Our study confirms these results and emphasizes the importance of NYHA class in the clinical assessment of cAVSD patients over the age of 40 years.

Despite a relatively young mean age of 43.7 years, at least one extracardiac comorbidity was already present in 93% of our patients. This is higher than reported for a large corresponding cohort of ACHD patients from the German National Register for Congenital Heart Defects [12]. This latter study included 4673 ACHD patients, and at least one comorbidity was present in 2882 patients (61.7%) altogether, and in 77.7% of patients over the age of 40 years [12]. As expected, complexity of CHD was associated with the presence as well as the number of comorbidities [12]. Considering that cAVSD is classified in the category of moderate or severe complexity in the Bethesda classification depending on the absence or presence of PAH, [13] this could provide an explanation for the higher proportion of patients with at least one comorbidity in our cohort. The proportion is also higher than in patients with Tetralogy of Fallot over the age of 40 years, which are classified in the moderate complexity group in the Bethesda classification. In a recent study, at least one acquired comorbidity was present in 66% of these patients [14]. However, in the same study, patients with a pulmonary atresia with a ventricular septal defect belonging to the severe complexity group, had in 88% of cases at least one extracardiac comorbidity in line with our results for cAVSD patients [14]. Therefore, awareness for extracardiac comorbidities and if possible preventive and therapeutic measures are important in our aging ACHD patients.

It is well described that cAVSD with an associated syndrome have a different clinical presentation than cAVSD patients without a syndrome [8]. Infants with AVSD and Down syndrome have different types of associated major non-cardiac malformations compared with infants without syndromes [3]. Furthermore, a better survival was reported for children with AVSD and Down syndrome at 1, 5, and 10 years of age [3]. This was not due to the presence of the Down syndrome per se, which was not a predictor of mortality but rather due to differences in AVSD complexity between children with Down syndrome and those without. In our study, the presence of a syndrome was also not predictive for the primary endpoint. Interestingly, renal disease, as well as gastrointestinal disorders, were more common in patients with a syndrome. This could be due to the fact that, in our cohort, patients with a syndrome were less likely to be operated on (52% vs. 72%) and more likely to have PAH (36% vs. 22%), as well as cyanosis (40% vs. 11%) than patients without a syndrome. The difference for the first two did not reach statistical significance, probably due to the small number of patients in each category. A possible explanation for this observation is that in the past it was a topic of discussion if cAVSD in Down syndrome patients should be repaired on the assumption that early surgery had a substantial risk with an unproven benefit [15]. An assumption which was later refuted [15]. A just decision, because when comparing patients with a corrected cAVSD vs. uncorrected patients in our study, in the latter group PAH (58% vs. 5%, *p* < 0.001), as well as renal diseases (42% vs. 8%, *p* = 0.009) were more common. Additionally, GI disorders were also more frequent (42% vs. 17%), but the difference did not reach a statistically significant difference (*p* = 0.07).

The detrimental effects of PAH and cyanosis are described as a multisystem disorder [16]. For example, it is well known that renal dysfunction has multifactorial causes and is a consequence of structural and functional abnormalities in cyanotic patients [17]. It also more common in cyanotic patients compared to acyanotic ACHD patients [18]. Additionally, it has a substantial impact on mortality [18]. The underlying mechanism remains a matter of debate [18]. Chronic hypoxia affects renal function directly and through the increased blood viscosity due to secondary erythrocytosis [18], which could lead to an increase in efferent glomerular arteriolar resistance, hydraulic pressure across the glomerulus and filtration fraction [18]. The consequence could be an increase in oncotic pressure in the post-glomerular vessels that perfuse the proximal tubules and promote reabsorption and fluid retention [18].

Furthermore, cholelithiasis, which is a consequence of the secondary erythrocytosis, is often encountered in cyanotic patients [16]. In a study from Japan, around 60% of patients with cyanotic CHD had a history of gallstones [16]. This could at least in part explain our observation regarding GI disorders. However, the difference between the corrected and uncorrected groups regarding the primary endpoint was not statistically significant (Figure 2), probably due to the small number of patients included.

We observed a significant difference regarding musculoskeletal disorders in the uncorrected group compared to the corrected group (32% vs. 4%, *p* = 0.02). Although gouty arthritis and hypertrophic osteoarthropathy are common skeletal complications in cyanotic patients [17], these were not frequent in our cohort. Therefore, the reasons for the observed difference are not clear.

The majority of studies on cAVSD focus on survival and reoperation, while little is known about ventricular function in the long term [8]. Reassuringly, in our study left ventricular systolic function was normal in all patients, while a more than mildly reduced right ventricular systolic function was found in only 3.4%. However, the right AV valve was more than mildly regurgitant in around 58% of patients, and the left AV valve in around 47%. Although current guidelines recommend the consideration of surgery in these circumstances [19], data for older adults like the patients in our cohort is lacking [20].

A limitation of our study is the small number of patients included. Although it is, to the best of our knowledge, the first to look at cAVSD patients over the age of 40 years, the number of patients is small. Future studies should address this issue by combining the data from large centers or using data from national registries. Furthermore, the retrospective design has some obvious limitations. In some patients, variables like the cause of death or echocardiographic data were missing. Results from exercise testing and laboratory analyses were often not available. Furthermore, while the study may inform us about the current cohort of cAVSD patients over the age of 40 years, future cohorts might differ with a more proactive surgical approach to cAVSD patients leading to a smaller proportion of unoperated patients and hence patients with PAH which should lead to a better outcome.

## 5. Conclusions

Significant morbidity and mortality is present in cAVSD patients over the age of 40 years. NYHA class is predictive for the combination of death from any cause and unplanned hospitalizations due to cardiac reasons.

## Figures and Tables

**Table 1:** Baseline characteristics with a comparison between patients with and without syndrome.

	All *n* (%)	Non-Syndromic *n* (%)	Syndrome *n* (%)	*p*
*n*	43	18	25	
Age (years)	43.7 ± 6.0	46.3 ± 8.0	41.9 ± 2.8	0.15
Female	26 (60.5)	10 (55.6)	16 (64.0)	0.75
History of				
Atrial arrhythmias	8 (18.6)	5 (27.8)	3 (12.0)	0.19
Ventricular arrhythmias	1 (2.3)	1 (5.6)	0 (0)	0.23
Cardiac surgery	26 (60.5)	13 (72.2)	13 (52.0)	0.19
Corrective	24 (55.8)	12 (66.7)	12 (48.0)	0.23
Cyanosis	12 (27.9)	2 (11.1)	10 (40.0)	**0.04**
NYHA class				0.67
I	28 (65.1)	12 (66.7)	16 (64.0)	
II	13 (30.2)	6 (33.3)	7 (28.0)	
III–IV	2 (4.7)	0	2 (8.0)	
Pulmonary arterial hypertension	13 (30.2)	4 (22.2)	9 (36.0)	0.33
Comorbidities (extracardiac), at least one	40 (93.0)	15 (83.3)	25 (100)	0.07
Musculoskeletal	7 (16.3)	2 (4.7)	5 (20.0)	0.44
Central nervous system	6 (14.0)	3 (16.7)	3 (12.0)	0.66
Renal	10 (23.3)	0 (0)	10 (40.0)	**0.002**
Genitourinary	7 (16.3)	4 (22.2)	3 (12.0)	0.37
GI incl. hepatobiliary	12 (27.9)	2 (11.1)	10 (40.0)	**0.037**
Lung	3 (7.0)	1 (5.6)	2 (8.0)	0.76
Eye/ear, nose and throat	6 (14.0)	1 (2.3)	5 (20.0)	0.18
Other	20 (46.5)	8 (44.4)	12 (48.0)	0.82
Hospitalizations	25 (58.1)	9 (50.0)	16 (64.0)	0.36
Unplanned cardiac hospitalizations	10 (23.3)	3 (16.7)	7 (28.0)	0.39
Death	4 (9.3)	1 (5.6)	3 (12.0)	0.47

**Table 2:** Baseline characteristics of patients with corrected vs. uncorrected cAVSD.

	All *n* (%)	Corrected *n* (%)	Uncorrected *n* (%)	*p*
*n*	43	24	19	
Age (years)	43.7 ± 6.0	46.5 ± 5.1	44.0 ± 7.0	0.49
Female,	26 (60.5)	13 (54.2)	13 (68.4)	0.34
History of				
Atrial arrhythmias	8 (18.6)	5 (20.8)	3 (15.8)	0.67
Ventricular arrhythmias	1 (2.3)	0	1 (5.3)	0.26
Cyanosis	12 (27.9)	0	12 (63.2)	**<0.001**
NYHA class				0.1
I	28 (65.1)	18 (75.0)	10 (52.6)	
II	13 (30.2)	6 (25.0)	7 (36.8)	
III–IV	2 (4.7)	0	2 (10.5)	
Pulmonary arterial hypertension	13 (30.2)	2 (4.7)	11 (57.9)	**<0.001**
Comorbidities (extracardiac), at least one	40 (93.0)	21 (87.5)	19 (100)	0.24
Musculoskeletal	7 (16.3)	1 (4.2)	6 (31.6)	**0.02**
Central nervous system	6 (14.0)	4 (16.7)	2 (10.5)	0.56
Renal	10 (23.3)	2 (8.3)	8 (42.1)	**0.009**
Genitourinary	7 (16.3)	3 (12.5)	4 (21.1)	0.45
GI incl. hepatobiliary	12 (27.9)	4 (16.7)	8 (42.1)	0.07
Lung	3 (7.0)	1 (4.2)	2 (10.5)	0.42
Eye/ear, nose and throat	6 (14.0)	3 (12.5)	3 (15.8)	0.76
Other	20 (46.5)	12 (50.0)	8 (42.1)	0.61
Hospitalizations	25 (58.1)	11 (45.8)	14 (73.7)	0.07
Unplanned cardiac hospitalizations	10 (23.3)	2 (8.3)	8 (42.1)	**0.01**
Death	4 (9.3)	1 (4.2)	3 (15.8)	0.19

**Table 3:** Univariate predictors for the primary endpoint.

Variable	HR (95% CI)	*p*
Age	1.04 (0.95–1.14)	0.40
Atrial arrhythmias	0.58 (0.11–2.94)	0.51
Extracardiac comorbidity	0.32 (0.04–2.85)	0.30
**NYHA class**	**1.96 (1.04–3.72)**	**<0.05**
Left AV-valve regurgitation	2.65 (0.75–9.28)	0.13
Right AV-valve regurgitation	2.24 (0.75–6.65)	0.15
PAH	0.51 (0.14–1.85)	0.31
Renal disease	2.82 (0.80–9.93)	0.11
Corrected	0.32 (0.07–1.54)	0.16
No previous cardiac surgery	2.17 (0.54–8.77)	0.28
Male	0.96 (0.27–3.40)	0.95
Non-syndromic	0.60 (0.15–2.33)	0.46
Cyanosis	3.49 (0.87–14.03)	0.08

HR indicates hazard ratio; NYHA indicates New York Heart Association; AV indicates atrioventricular; PAH indicates pulmonary arterial hypertension.

## Data Availability

The data associated with this paper are not publicly available.
